# Assessing Variations in Host Resistance to *Fusarium oxysporum* f sp. *cubense* Race 4 in *Musa* Species, With a Focus on the Subtropical Race 4

**DOI:** 10.3389/fmicb.2019.01062

**Published:** 2019-05-15

**Authors:** Andrew Chen, Jiaman Sun, Andrea Matthews, Liz Armas-Egas, Ning Chen, Sharon Hamill, Sharl Mintoff, Lucy T. T. Tran-Nguyen, Jaqueline Batley, Elizabeth A. B. Aitken

**Affiliations:** ^1^School of Agriculture and Food Science, The University of Queensland, Brisbane, QLD, Australia; ^2^Guangxi Crop Genetic Improvement and Biotechnology Key Lab, Guangxi Academy of Agricultural Sciences, Nanning, China; ^3^Department of Agriculture and Fisheries, Maroochy Research Facility, Nambour, QLD, Australia; ^4^Department of Primary Industry and Resources, Northern Territory Government, Darwin, NT, Australia; ^5^School of Biological Sciences, The University of Western Australia, Perth, WA, Australia

**Keywords:** *Fusarium* wilt, banana, *Musa acuminata* ssp. *malaccensis*, green fluorescent protein, *Fusarium oxysporum* f. sp. *cubense*

## Abstract

*Fusarium oxysporum* f. sp. *cubense* (*Foc*) has severely curtailed banana production in the tropical regions of the world. The tropical race 4 (TR4) of *Foc* was detected in Australia in the 1990s and it is virulent to all Cavendish type banana cultivars, which represents the majority of banana production in Australia. Genetic resistance to *Foc* race 4 is urgently needed. To characterize sources of resistance, we have assessed the *Foc* resistance response of 34 *Musa* cultivars with plants grown under controlled settings. Amongst diploid banana cultivars carrying the AA genome, resistance is found in *Musa acuminata* sub-species including *malaccensis* ‘Pahang’ and *burmannica* ‘Calcutta4.’ In the polyploid group, the hybrids such as ‘FHIA-18’ and ‘FHIA-25’ are highly resistant against both *Foc*-TR4 and subtropical race 4 (*Foc*-STR4). Interestingly, ‘FHIA-2’ and ‘CAM020’ appear to be resistant to *Foc*-TR4 but susceptible to *Foc*-STR4, suggesting potential differences in the resistance mechanisms against the different race 4 strains. Using a GFP tagged *Foc*-STR4 strain challenged onto both resistant and susceptible *M. a. malaccensis* lines, a high inoculum dosage rapidly induced vascular wilt in the susceptible *M. a. malaccensis* lines at 2.5 weeks. This was associated with an accumulation of micro-conidia in the rhizome and the movement of the fungus through the xylem vessels. In contrast, the fungal movement was restrained in the rhizome of the resistant *M. a. malaccensis* lines and no sporulation was observed. Overall, this research suggests that the resistance response is dependent to an extent on inoculum dosage and that the plant host’s response, in the rhizome, plays an important role in inhibiting the fungus from spreading to the rest of the plant. Identifying race 4 resistant accessions can help to understand mechanisms of resistance and provide banana breeders with the genetic resources to integrate resistance genes into commercial varieties.

## Introduction

Banana and plantain (*Musa* spp.) serve as important sources of staple food and fruit around the world and collectively are considered the world’s leading fruit crop, with a production value reaching over 100 million tons per annum (FAO, 2019). As a staple food, banana is an important export commodity in Africa and Asia, ensuring food security for millions of people (Aurore et al., 2009). One of the major constraints in the global production of banana is the disease, *Fusarium* wilt. It is also known as Panama disease, which is caused by *Fusarium oxysporum* f. sp. *cubense* (*Foc*) (Ploetz, 2006).

*Foc* gains entry into the plant host via roots. Once inside, it colonizes the rhizome and travels up the pseudostem, where it blocks the water-conducting xylem vessels and thus prevents the transport of water and nutrients to the aerial parts of the plant. External symptoms of *Fusarium* wilt start with the yellowing and wilting of the older leaves and progress to the younger leaves until the plant dies. Internally, the plants show brown discoloration and necrosis of xylem vessels in the rhizomes and stems. The disease incidence varies depending on the cultivar, the environment and the level of inoculum, but can extend to total crop loss in heavily infested fields (Moore et al., 2001).

*Foc* is a soil-borne pathogen that produces chlamydospores enabling its survival in the soil in the absence of the host. It is also known to survive on weed hosts in a non-pathogenic manner (Hennessy et al., 2005). Once the soil is infested with *Foc*, it generally becomes unsuitable for replanting for many years thereafter (Stover, 1990). Furthermore, unlike the black Sigatoka leaf spot caused by *Pseudocercospora fijiensis*, the other major fungal pathogen affecting the banana industry worldwide, *Fusarium* wilt cannot be controlled by fungicides (Ploetz, 2006).

Outbreaks of *Fusarium* wilt decimated a banana industry primarily based on the cultivar ‘Gros Michel’ (AAA) in Central America in the 1950s. The pathogen was subsequently named *Foc* race 1, and the outbreak forced the industry to shift production to the *Foc* race 1 resistant cultivars of the Cavendish (AAA) subgroup (Stover, 1990). Cavendish now accounts for >40% of world banana production, with export markets amounting to 15% of the total production (FAO, 2019).

Within the *forma specialis* (f. sp.) *cubense*, which include all isolates pathogenic to *Musa* spp. (Snyder and Hansen, 1940), there is significant pathogenic variation; members of this f. sp. are divided into four races based on their host range (Stover, 1990). Race 1 is pathogenic to ‘Gros Michel’ (AAA) and a range of other cultivars carrying the AAB genome. Race 2 targets those race 1-susceptible cultivars, as well as the hybrid triploid ‘Bluggoe’ (AAB). Race 3 affects *Heliconia* species and is no longer considered to be part of the *cubense* race structure. Race 4 is pathogenic to all race 1 and race 2 susceptible cultivars plus the Cavendish subgroup (AAA) (Su et al., 1986). Race 4 is further divided into two groups: tropical (TR4) and subtropical (STR4) race 4. *Foc*-STR4 isolates cause disease in Cavendish in the subtropics, whereas *Foc*-TR4 isolates are pathogenic to Cavendish under both tropical and subtropical conditions (Buddenhagen, 2009).

*Foc* isolates can also be grouped according to vegetative compatibility, which is the ability of an isolate to anastomose and form a stable heterokaryon (Moore et al., 1993). Isolates that are vegetatively compatible with one another form a VCG and typically share common biological, physiological and pathological traits (Caten and Jinks, 1966). At least 21 different VCGs of *Foc* have been characterized, with the majority of the groups present in Asia, where the pathogen is thought to have evolved (Fourie et al., 2009). *Foc*-TR4 isolates are designated as VCG 01213 (or VCG 01216, which is a different designation for the same VCG) (Bentley et al., 1998). *Foc*-STR4 isolates are designated as VCGs 0120, 0121, 0122, 0129, and 01211 (Buddenhagen, 2009). Since its appearance in Southeast Asia in the 1990s, *Foc*-TR4 (VCGs 01213 or 01216) has caused severe damage to Cavendish plantations in Malaysia, Indonesia, China, the Philippines and the Northern Territory and Queensland in Australia as well as Mozambique (Ploetz, 2006, 2015; Buddenhagen, 2009). The virulence and epidemic nature of *Foc*-TR4 is due to its potent pathogenicity and wide host range within the genus *Musa*.

Genetic resistance can offer a long-term means of control of *Fusarium* wilt, but no truly *Foc*-TR4 resistant commercially viable cultivar is available. Efforts have been made to identify *Foc*-TR4 resistant cultivars (Zhang et al., 2018; Zuo et al., 2018) but transferring these favorable alleles into a commercially viable cultivar with good agronomic traits has been a challenge (Dita et al., 2018). Somaclonal variants of Cavendish known as the ‘giant Cavendish tissue culture variants’ or ‘GCTCVs’ have been generated in Taiwan through tissue culturing and field trials and have shown that they possess some improved level of tolerance to *Foc*-TR4 (Hwang and Ko, 2004). However, these lines vary in their levels of tolerance and are considered by some to contain undesirable agronomic traits (Ploetz, 2015). Therefore, genotypic screening of cultivated and wild germplasm is extremely important in characterizing existing or novel sources of *Foc* race 4 resistance, which could assist phenotypic selection in breeding programs or facilitate the isolation of gene(s) underlying resistance for the purpose of engineering resistance in commercial cultivars via gene technologies.

In cultivated banana, four genome types have been identified and they include *M. acuminata* (A), *M. balbisiana* (B), *M. schizocarpa* (S) and those of the *Australimusa* section (T) (D’Hont et al., 2000). So far, most of the cultivated banana plants are diploid or triploid, originating from intra- or inter-specific hybridizations between the diploid A and the B genomes (Perrier et al., 2011). Most of the commercial cultivars are seedless and produce fruit by parthenocarpy, resulting in limited selection process and mono-culture productions. Most of these cultivars are therefore susceptible to biotic and abiotic stress, indicating a limited gene pool (D’Hont et al., 2012). Wild relatives of commercial varieties and other cultivated diploids such as ‘Pisang Jari Buaya,’ produce seeds and are considered good sources of genetic diversity worthy of exploration for improving resistance to *Fusarium* wilt. Indeed, *resistance gene analog* 2 (*RGA*2) was isolated from *Musa acuminata* ssp. *malaccensis* and has recently been shown to confer resistance to TR4 in field trials conducted in Australia (Peraza-Echeverria et al., 2008; Dale et al., 2017).

In this study, we assessed the resistance level of 34 banana cultivars against *Foc*-TR4 and *Foc*-STR4. These include diploids (AA and BB groups, and wild relatives) and intra- or inter-specific hybrids such as Cavendish banana ‘GCTCVs’ and plantains from the FHIA. We used a pot based bio-assay method to assess the level of resistance in each accession grown under glasshouse conditions. We characterized several *Foc* race 4 resistant diploids as a source of resistance; these sources may have already been incorporated into the resistant hybrids through conventional breeding. We also show that resistance levels vary amongst the genotypes and resistance is likely of quantitative nature across a spectrum. The level of observed resistance under controlled environmental conditions can be determined by factors such as inoculum dosage, maturity of plants and inoculation technique. Using a GFP tagged *Foc*-STR4 strain, we show that the rhizome plays an important role as a barrier to the pathogen preventing it from migrating toward the rest of the plant. We identified a diploid wild relative that exhibits a strong resistance response to *Foc* manifested by reduced fungal penetration in root cells and containment of the fungi in the rhizome. This is therefore, a potential novel source of resistance to *Foc* race 4 types.

## Materials and Methods

### *Fusarium* Strains

The monoconidial *Foc*-TR4 strain (NTPDc 35673) was originally collected from the Coastal Plains Research Farm at in the Northern Territory, Australia in the early 2000s. In 2016, VCG testing performed on this strain by Department of Agriculture and Fisheries (DAF, Nambour, QLD, Australia) confirmed that it is VCG 01213/16.

Three monoconidial isolates of *Foc* VCG0120 were obtained from the Queensland Plant Pathology Herbarium (BRIP; 63488, 43781, and 42331) at the Queensland Department of Agriculture and Fisheries. *Foc* BRIP 23598 from VCG 0120 was previously transformed with GFP (Henceforth, GFP-*Foc*-STR4) and stored at -80°C (Forsyth, 2006).

All *Foc* isolates were cultured on half-strength potato dextrose agar (PDA) Difco (Becton, Dickson and Co., Sparks, MD, United States) for 7 days at 25°C. The GFP-*Foc*-STR4 isolate was regenerated on the same media but with the addition of 50 mg L^-1^ of hygromycin B.

### Preparation of Millet Inoculum

To prepare millet grain for *Foc*-STR4 inoculum, millet (*Pennisetum glaucum*) seed was washed in tap water, covered with distilled water, and then soaked overnight in a suitable container. Excess water was drained from the seed using a sieve, then the grain was rinsed a second time in distilled water to remove leached carbohydrate. The grain was then placed into Erlenmeyer flasks or other suitable containers and autoclaved twice for 20 min at 120°C on consecutive days. Once cooled, the grain was inoculated with approximately five 1 cm squared mycelial plugs cut from *Foc* cultures grown on half strength PDA. Flasks were shaken daily to distribute *Foc* evenly. When the millet was fully colonized by the *Fusarium*, it was used as plant inoculum.

The *Foc*-TR4 inoculum was prepared with the following modifications to allow an up-scale. Batches of 1.5 kg of millet were placed in small autoclave bags. After the addition of 500 mL of RO water, the bags were sealed and autoclaved twice over consecutive days. Half of a PDA plate containing *Foc*-TR4 was added to each bag of autoclaved millet. The bags were shaken every second day.

For GFP studies, four to five mycelial agar plugs of GFP-*Foc*-STR4 were added to half strength potato dextrose broth (PDB, Difco) containing 50 mg L^-1^ hygromycin B and shaken gently at 28°C for 5 days. The culture was processed using a previously described method (Li et al., 2011) to extract viable spores and a final concentration of 2 × 10^6^ conidia mL^-1^ was prepared for root dipping. All work involving GFP transformed strains of *Foc*, was conducted under conditions of an NLRD (notifiable low risk dealing) permit according to the Office and Gene Technology Regulator, Australia.

### Plant Materials and Growth

Wild relatives of the cultivated banana are known to harbor resistance against *Foc*. So far, field studies have identified resistant genotypes against *Foc* race 4 and those include diploids (AA genome) from *Musa accuminata* ssp. *malaccensis*, *Musa accuminata* ssp. *Banksii*, and *Musa accuminata* ssp. *burmannica* (Table 1). In this study, we selected 34 accessions of various ploidy levels to assess their resistance response to *Foc*-STR4 and *Foc*-TR4 in pot trials (Table 1). The diploids used include *M. a. malaccensis* from Sumatra, Indonesia (‘Ma846,’ ‘Ma848,’ ‘Ma850,’ ‘Ma851,’ ‘Ma852’) and from Malaysia, ‘Pahang’ and *M. a. malaccensis* of ITC0250. We also examined ‘Calcutta4’ (*M. a. burmannica*) and ‘Pisang Jari Buaya’ (AA) that have been shown to carry race 4 resistance in field trials (Table 1). Other diploid cultivars tested include ‘SH3217,’ ‘SH3362,’ and ‘SH3142’ that have been used extensively in breeding programs such as FHIA. The hybrids with polyploid genomes tested include ‘FHIA-1,’ ‘FHIA-2,’ ‘FHIA-3,’ ‘FHIA-18,’ ‘FHIA-23,’ ‘FHIA-25,’ ‘FHIA-26.’ We also looked at the two ‘GCTCVs’ lines (119 and 218) of Taiwan origin that exhibited improved tolerance against *Foc*-TR4 in the field (Hwang and Ko, 2004). Lines from breeding programs were also examined. These include ‘M61 Guadeloupe,’ an elite bred diploid from the Jamaican breeding program and ‘CAM020’ which is an F_1_ individual from a cross between ‘Calcutta 4’ and ‘Banksii Madang.’ It is part of the ‘AFCAM20’ population developed by INIBAP (International Network for the Improvement of Banana and Plantain).

**Table 1:** List of genotypes presented in this study, and the available corresponding field studies that determined their resistance responses against *Foc* race 4 types.

Genotype name	Genome	Origin	ITC No	Field resistance	Present study
Ma851	AA	*malaccensis*	–	R^T^(a)	R^S^
Ma852	AA	*malaccensis*	–	R^T^(a)	R^S^
Calcutta4-IV9	AA	*burmannica*	–	R^T^(b)	R^T^, R^S^
Pahang	AA	*malaccensis*	ITC0609	R^T^(b)	R^T^, R^S^
SH-3217	AA	Hybrid	–	–	R^S^
SH-3362	AA	Hybrid	–	–	R^T^, R^S^
SH-3142	AA	Hybrid	ITC0425	SS^T^(b)	R^T^, R^S^
Madang Gaudelope	*M. acuminata*	*banksii*	–	–	R^T^, R^S^
FHIA-1	AAAB	Hybrid	ITC0504	SS^T^(a), S^T^(c), R^S^(d)	R^T^, R^S^
FHIA-25	AAB	Hybrid	ITC1418	R^T^(a), R^T^(b)	R^T^, R^S^
GCTCV-119	AAA	Cavendish	ITC1282	SS^T^(a), R^T^(e)	R^T^, R^S^
Ma850	AA	*malaccensis*	–	R^T^(a)	R^T^, R^S^
Pisang Jari Buaya	AA	c.v.	–	SS^T^(a), R^T^(b)	R^T^, R^S^
Calcutta-4	AA	*burmannica*	ITC0249	R^T^(b)	R^T^, R^S^
FHIA-18	AAAB	Hybrid	–	SS^T^(a), R^S^(d)	R^T^, R^S^
Ma250	AA	*balaccensis*	ITC0250	–	R^S^
Pisang bangkahulu	AA	c.v.	ITC0689	–	R^S^
M61 Gaudelope	–	–	–	–	R^T^, SS^S^
GCTCV-218	AAA	Cavendish	ITC1597	S^T^(a), R^T^(e)	SS^S^
Williams	AAA	Cavendish	–	HS^T^(a), S^T^(b), MS^S^(d)	S^T^, SS^S^,
Khae (Phrae)	*M. siamea*	*siamea*	ITC0660	–	SS^S^
Pisang raja	AAB	c.v.	ITC0243	R^T^(b)	MS^S^
Pisang madu	AA	c.v.	ITC0258	SS^T^(b)	MS^S^
FHIA-23	AAAA	Hybrid	ITC1265	HS^T^(a), S^T^(b)	SS^T^, S^S^
Ma846	AA	*malaccensis*	-	S^T^(a)	S^S^
FHIA-2	AAAA	Hybrid	ITC0505	HS^S^(d)	R^T^, S^S^
*Balbisiana*	BB	*balbisiana*	-	MS^T^(c)	S^S^
*Musa zebrina*	AA	*zebrina*	ITC1177	SS^T^(b)	S^S^
FHIA-3	AABB	Hybrid	ITC0506	S^T^(b)	SS^T^, S^S^
CAM-020	AAA	Cavendish	–	–	R^T^, S^S^
Ma848	AA	*malaccensis*	–	S^T^(a)	S^T^, S^S^
FHIA-26	AABB	Hybrid	1422	–	S^T^
Lady Finger	AAB	c.v.	–	HS^T^(a), HS^S^(d)	S^T^
Pisang Gajih Merah	ABB	Hybrid	Indonesia	–	SS^T^

*R, SS, MS, S, and HS abbreviate resistant, slightly susceptible, moderately susceptible, susceptible, and highly susceptible over-all wilting symptoms from the field studies, respectively. Symbols ^*S*^ and ^*T*^ indicates *Foc* sub-tropical race 4 and *Foc* tropical race 4, respectively. All except the study performed by Walduck and Daly (2007), used naturally infested soil types. The latter study used additional susceptible plants as inoculum between the trial plants. References are listed in brackets and are abbreviated using the following letters, a = Walduck and Daly, 2007; b = Zuo et al., 2018; c = Li et al., 2015; d = Smith et al., 2014; e = Hwang and Ko, 2004. In the present study, resistance responses are categorized according to the mean scores of the rhizome discoloration per cultivar; resistant (R) = 1 to 3; slightly susceptible (SS) ≥ 3 and ≤ 4; moderately susceptible (MS) ≥ 4 to ≤ 5; susceptible (S) ≥ 5*.

Banana germplasm was kindly supplied by the Maroochy Research Facility at the Queensland Department of Agriculture and Fisheries. Germplasm is listed in Table 1 and those with known corresponding ITC numbers are indicated. Six to eight clones of each accession from Table 1 were micro-propagated and maintained *in vitro* as per previous study (Smith and Hamill, 1993). Seedlings approximately 10 cm in height with three to four true leaves were de-flasked into 30 well seedling trays to harden off in the laboratory under constant LED lights. The seedlings were transferred into 200 mm diameter pots in designated spaces for disease screening. *Foc*-TR4 is strictly quarantined in Australia and pathogen screening was instead carried out in a screen house at the Coastal Plains Research Station (Department of Primary Industry and Resources, Northern Territory) in Darwin, Australia. The average daily temperature in Darwin ranged from 30 to 35°C during the August to November months when the pot trials were conducted. *Foc*-STR4 screening was performed in the University of Queensland (UQ) glasshouse facility with temperature control set at 25 to 28°C. The potting mix contained 70% composted pink bark and 30% coco peat at a pH range of 5.5 to 6.5.

### Plant Inoculation

Once the pseudostem of most plants reached 30 cm in height and each plant carried five to six true leaves, the plants were inoculated with either *Foc*-STR4 or *Foc*-TR4 infested millet or spores of GFP-*Foc*-STR4 at pre-determined concentrations. A total of 40 g of *Foc*-STR4 or *Foc*-TR4 infested millet was used as inoculum per 200 mm diameter pot. For inoculation, plants were briefly removed from the pots and 20 g of millet was added to the bottom of the pots before plants were re-potted. Another 20 g of millet was then spread evenly on the surface soil layer and finally covered with additional soil.

### Assessment of Symptoms

Plants inoculated with *Foc*-STR4 or *Foc*-TR4 infested millet were scored for internal symptoms 10 to 12 weeks post-inoculation. The rhizome was vertically cut in halves to score the extent of discoloration. The rating scale for rhizome discoloration ranges from 1 to 8, with 1 being no discoloration and 8 indicating the entire rhizome is discolored and the plant dead (Mak et al., 2004). The mean score from three to eight plants was used to quantify the plant response.

### Confocal Microscopy

Confocal microscopy was used to visualize GFP-*Foc*-STR4 in two separate experiments. Firstly, ‘Ma851’ self-derived progeny ‘p168’ (resistant) and ‘p248’ (susceptible) were examined for the presence of GFP-*Foc*-STR4 in the rhizome. Plants were inoculated with GFP-*Foc*-STR4 infested millet. Assessment was performed at 3 months post-inoculation. In the second experiment, the parent ‘Ma851’ (resistant) and ‘Ma848’ (susceptible) were used. Plant roots were dipped in a GFP-*Foc*-STR4 spore suspension containing 2 × 10^-6^ spores per mL for 2 h. Inoculated plants were then re-potted using soil containing 50,000 GFP-*Foc*-STR4 conidia per g of soil which is 5–10-fold of what is typically used in this type of assay (Li et al., 2012). The pathogen infection processes in these plants were observed at 1, 2, 3, 4, 7, and 14 dpi.

Transverse and longitudinal sections were hand-prepared to visualize pathogen development on the root surface and in the vascular bundles and the corms. Sliced sections were counter-stained with propidium iodide (PI, Sigma Aldrich) for 5 min at a concentration of 10 μg mL^-1^. A Zeiss 700 laser scanning microscope was used to visualize and acquire the confocal images. The GFP and the PI were detected using the 488 and 555 nm lasers, respectively. Z-stack acquisition mode was used to obtain 3D images consisting of 10–20 optical slices taken at intervals of 1–5 μm. T-PMT (transmission detector setting) was used to obtain the light images of the sectioned tissue.

### Koch’s Postulates

Primary isolations were performed on three resistant (104A, 3A, 18A) and one susceptible (96B) self-derived progeny of ‘Ma851’ to investigate the presence or absence of GFP-*Foc*-STR4. Fungal strains were isolated 3 months post-inoculation with GFP-*Foc*-STR4 infected millet. Five regions were isolated per plant, which included the upper stem just below the first leaf petiole, mid-point of the stem, stem just above the rhizome, the central cylinder of the rhizome and the outer layer of the rhizome connected to the cortex. Four pieces of tissues were isolated in each region. These sectioned pieces were surface sterilized for 1 min in 1% hypochlorite solution, then rinsed twice in distilled water for 30 s each time. Each piece was air-dried and plated onto water agar plates containing 100 mg L^-1^ streptomycin for 5 days at 24°C. Segments showing *Fusarium*-like growth under a dissecting microscope were further isolated and then hyphal tipped to generate monoconidial isolates on half strength potato dextrose agar (PDA) plates containing 50 mg per L hygromycin B. Each isolate was then transferred to 20 mL of potato dextrose broth containing 50 mg L^-1^ hygromycin B (PDB) and incubated at 26°C on a platform rotating at 160 rpm for 4 days. GFP fluorescence of the isolates was visualized under Zeiss 700 confocal microscope to confirm the presence of the GFP-*Foc*-STR4 strain.

## Results

### *Foc*-STR4 Screening Trial

**Figure 1:**
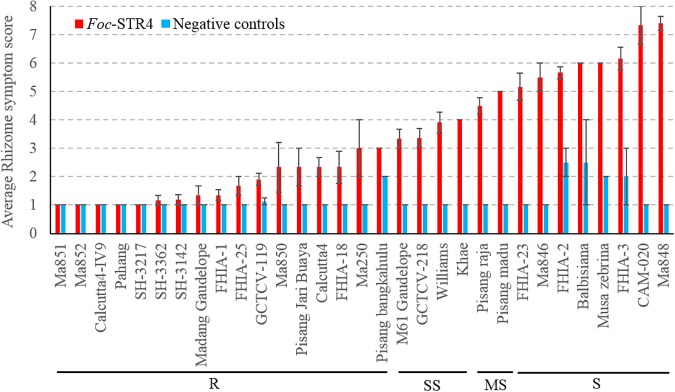
Sensitivity of different banana genotypes to *Foc*-STR4 in glasshouse pot trials. Internal symptoms were assessed by using a scoring system based on the percentage of rhizome discoloration (Mak et al., 2004). Millet was inoculated separately using three *Foc*-STR4 isolates BRIP63488, BRIP43781, and BRIP42331 of VCG 0120. Fully colonized millet from each strain was mixed in equal amounts and 40 g of inoculum was applied to each 200 mm diameter pot. Clean autoclaved millet was used as negative controls. Error bars indicate standard deviations of the mean derived from three to eight individual plants. Genotypes that have rhizome symptom scores between 1 and 3 (≤5% rhizome discoloration) are categorized in the resistant group (R); those with a score between 3 and 4 (>5 to ≤20% discoloration) are categorized in the slightly susceptible group (SS); those that score between 4 and 5 (>21 to ≤50%) are categorized as susceptibles (MS); those that score greater than 5 (>50%) is categorized as susceptible (S).

Thirty-four genotypes were tested for their response to *Foc*-STR4 under glasshouse conditions (Table 1). Following inoculation, the rhizome discoloration was scored using a predetermined scale (Mak et al., 2004) which is presented in Table 2. A rhizome score of 1 or 2 indicates no discoloration in the rhizome 3 months after inoculation and is associated with the AA diploids ‘Ma851,’ ‘Ma852,’ ‘Calcutta4-IV9,’ ‘SH-3217,’ ‘SH-3362,’ ‘SH-3142,’ as well as the hybrids ‘FHIA-1’ (Gold Finger), ‘FHIA-25’ and ‘GCTCV-119’ (Figure 1). Accessions that had a clear rhizome but had little or some discoloration around the junctions of roots joining the rhizome are categorized by a score of 2 to 3 (≤5% discoloration) and include the diploids ‘Ma850,’ ‘Pisang Jari Buaya,’ ‘Calcutta-4,’ ‘FHIA-18,’ ‘Ma250,’ and ‘Pisang bangkahulu.’ In this group, some of these lines, namely ‘Ma850,’ ‘Pisang Jari Buaya,’ ‘FHIA-18,’ and ‘Ma250’ had large phenotypic variation in the degree of rhizome discoloration amongst individual clones, possibly due to somaclonal variations of genetic or epigenetic origin (Figure 1 and Supplementary Figure 1). These lines also exhibited a good level of resistance against *Foc*-TR4 in previous field studies (Table 1). Accessions that display up to 20% rhizome discoloration fall into the slightly susceptible (SS) group. These include‘ ‘M61 Gaudelope,’ ‘GCTCV218,’ ‘Williams,’ ‘Khae’ (Figure 1). ‘Pisang raja’ and ‘Pisang madu’ showed discoloration in the 21 to 50% range and are hence considered moderately susceptible (Figure 1). The accessions that showed more than 50% discoloration are considered susceptible (S) to *Foc*-STR4. These include ‘FHIA-2,’ ‘FHIA-23,’ ‘Ma846,’ *Musa balbisiana*, *Musa accuminata* ssp. *zebrina*, ‘FHIA-3,’ ‘CAM020,’ and ‘Ma848’ (Figure 1). ‘CAM-020’ and ‘Ma-848’ were extremely susceptible to *Foc*-STR4; half of the clonal plants were dead at the time of assessment.

**Table 2:** Rhizome discoloration index as per previously described (Mak et al., 2004).

Discoloration index	Description
1	No discoloration of the tissue in the stelar region of the rhizome and the surrounding region.
2	No discoloration of the stellar region of the rhizome. Discoloration at the junctions of root and rhizome.
3	Trace up to 5% of the stellar region discolored
4	6 to 20% of the stellar region discolored.
5	21 to 50% of the stellar region discolored.
6	More than 50% of the stellar region discolored.
7	The entire rhizome stele is discolored.
8	The plant is completely dead.

### *Foc*-TR4 Screening Trial

**Figure 2:**
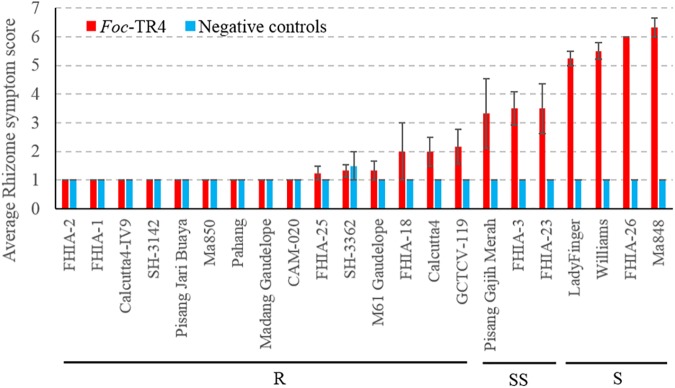
Sensitivity of different banana genotypes to *Foc*-TR4 in the shade house pot trial. Internal symptoms were assessed by using a scoring system based on the percentage of rhizome discoloration (Mak et al., 2004). Millet infested with *Foc*-TR4 strains carrying VCG 01213/16 was used as the inoculum. For each 200 mm diameter pot, 40 g of the inoculum was applied. Clean autoclaved millet grains were used in uninoculated control treatments. Error bars indicate standard deviations of the mean derived from three to eight individual plants. Abbreviations and categories of resistance are the same as per Figure 1.

Due to logistical constraints, only a subset of accessions was selected for testing against *Foc*-TR4 in the Northern Territory, Australia under shade house conditions. At the time of the assessment, the susceptible plants did not show noticeable external symptoms, however, internal symptoms were present. Internally, the diploids *M. a. malaccensis* (AA) ‘Pahang’ and ‘Ma850’ displayed clean rhizomes that suggests a high level of resistance against *Foc*-TR4 (Figure 2). Other accessions that produced resistant (R) rhizome phenotypes include ‘FHIA-1,’ ‘FHIA-2,’ ‘FHIA-18,’ ‘FHIA-25,’ ‘Calcutta4,’ ‘Calcutta4-IV9,’ ‘SH-3142,’ ‘SH-3362,’ ‘Pisang Jari Buaya,’ ‘Madang Guadelope,’ ‘M61 Guadelope,’ ‘CAM-020,’ and ‘GCTCV-119’ (Figure 2). The SS group includes ‘Pisang Gajih Merah,’ ‘FHIA-3,’ and ‘FHIA-23.’ All three cultivars displayed a high level of phenotypic variation in clonal plants, possibly owing to somaclonal variations (Figure 2 and Supplementary Figure 2). ‘Lady Finger,’ ‘Williams,’ ‘FHIA-26,’ and ‘Ma848’ all showed severe discoloration in their rhizomes and hence are considered as susceptible (S) to *Foc*-TR4 (Figure 2).

### Comparison Between *Foc*-STR4 and *Foc*-TR4 Induced Responses

Most accessions showed consistent responses between the two race 4 types (Table 1). ‘Williams,’ which belongs to the Cavendish subgroup, is the dominant variety used in commercial production in Australia. In our study, it was highly susceptible to *Foc*-TR4 but was only partially susceptible against *Foc*-STR4 when tested under glasshouse conditions (Figure 1, 2). Our results are consistent with the resistance response of ‘Williams’ to race 4 types detected in the field (Table 1). In these studies, Cavendish types (AAA) have generally shown high susceptibility against *Foc* race 4 but improved resistance has been detected in Cavendish variants in the field (Bhagwat and Duncan, 1998; Hwang and Ko, 2004). The giant Cavendish type somatic mutant ‘GCTVC119’ showed resistance against both *Foc*-STR4 and *Foc*-TR4 in our study (Figure 1, 2). However, the variant ‘GCTCV-218’ showed slight susceptibility against *Foc*-STR4. *Foc*-TR4 resistance levels of ‘GCTCV-218’ does not appear to be consistent as shown by field results (Table 1), possibly suggesting the presence of genotype-environment interactions at the different trial locations. ‘M61 Gaudelope,’ ‘FHIA-3,’ ‘FHIA-23,’ ‘Pisang raja’ all showed a relatively higher level of susceptibility to *Foc*-STR4 than *Foc*-TR4 (Table 1). Interestingly, ‘FHIA-2’ and ‘CAM-020’ were both resistant to *Foc*-TR4 but showed highly susceptible rhizome phenotypes to *Foc*-STR4 (Figure 1, 2).

### Pathogen Infection Process

**Figure 3:**
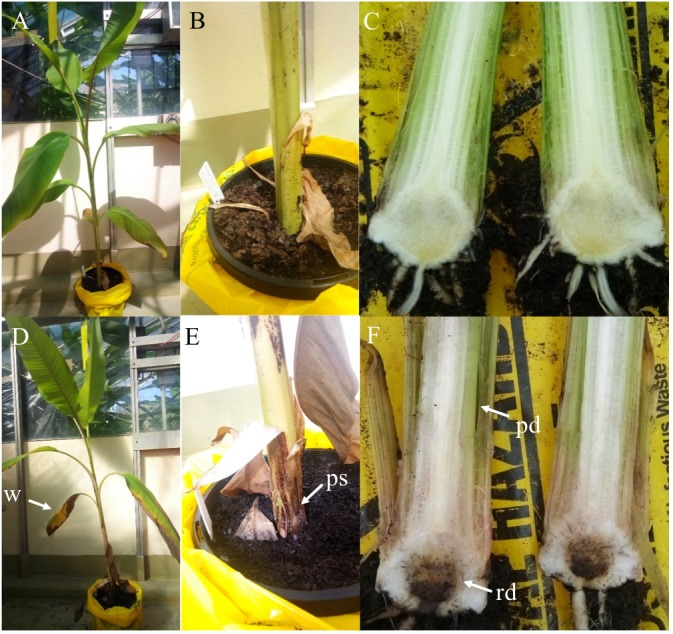
Self-derived F_2_ progeny of the resistant parental line ‘Ma851’ showing contrasting resistance response against GFP-*Foc*-STR4. Plants were inoculated with GFP-*Foc*-STR4 infested millet and photographs were taken 3 months after inoculation. **(A)** The resistant progeny plant ‘p168’ showing no leaf wilting symptoms and was relatively healthy. **(B)** The base of the pseudostem just above the soil showing no signs of stem splitting, which is a manifestation of the wilting disease. **(C)** The rhizome of ‘p168,’ cut in half longitudinally, showed no traces of brown discoloration in the lower and center regions. **(D)** The susceptible progeny plant ‘p248’ derived from the same F_2_ cross displayed wilting of the old leaves. **(E)** Stem splitting at the base of ‘p248’ was clearly visible just above the soil level. **(F)** The rhizome of ‘p248’ rhizome developed extensive brown discoloration which is typically associated with high *Foc* susceptibility. Arrows indicate the sites of external and internal symptoms at the time of assessment. w = wilting of old leaves, ps = pseudostem splitting, rd = rhizome discoloration, pd = pseudostem discoloration.

**Figure 4:**
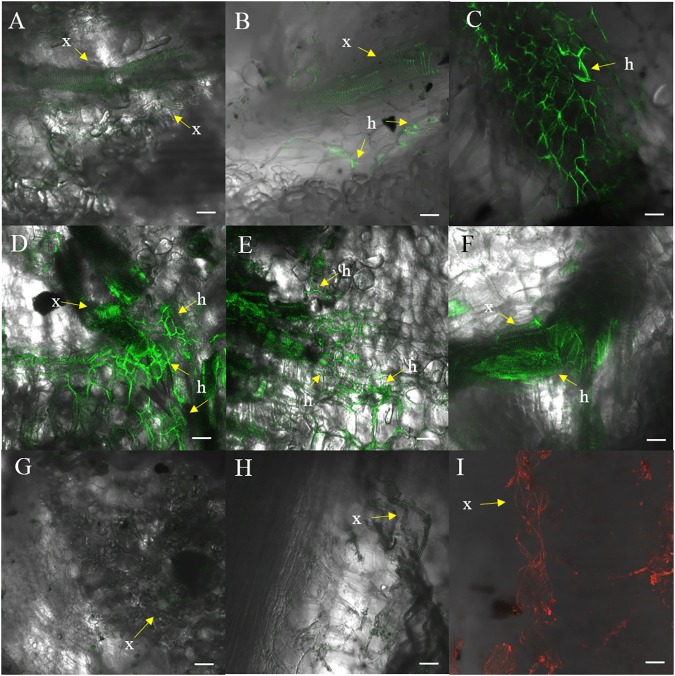
Confocal images of ‘p168’ (resistant) and ‘p248’ (susceptible) at 3 months post-inoculation performed using millet and GFP-*Foc*-STR4. **(A)** Xylem perforation plates observed containing GFP observed in the lower region of the ‘p168’ rhizome. **(B)** GFP linked to the perforation plates and the hyphae structure in the lower region of the rhizome bordering the cortex cells in ‘p168.’ **(C)** Presence of GFP tagged mycelial networks in the lower region of the rhizome in ‘p168.’ **(D)** A cross section of the lower region of the ‘p248’ rhizome showing xylem perforation plates and the expansion of mycelial networks associated with GFP. **(E)** A longitudinal section of the lower region of the ‘p248’ rhizome showing GFP associated with perforation plates and the expanded mycelial networks. **(F)** Mycelial structures establishing near the boundary between the central region of the rhizome and the cortex area in ‘p248.’ **(G)** Confocal showing the absence of GFP in the lower rhizome of the un-inoculated control ‘p4-19’ derived from ‘Ma851.’ **(H)** Xylem perforation plates with no associated fluorescence in the non-inoculated controls of ‘p4-19.’ **(I)** Xylem perforation plates visualized under a 555 nm laser using non-inoculated ‘p4-19’ rhizome stained with propidium iodide. GFP was visualized using the 488 nm laser. Light images are created using the T-PMT (transmitted light detector) setting. Scaled bars represent a 50 μm unit. Arrows indicate the presence of xylem vessels (x) and hyphae (h).

Fluorescence produced by GFP was visualized to assess the extent of the spread of the GFP-*Foc*-STR4 inside the rhizome of plants that had been inoculated with millet infested with the fungus. Resistant ‘p168’ and susceptible ‘p248’ progeny of ‘Ma851’ were compared. ‘P168’ and ‘p248’ were the self-pollinated progeny between the clonal plants of the parent ‘Ma851.’ Previously, segregation analysis using the self-derived progeny of ‘Ma851’ showed that they segregated for both *Foc* race 4 type resistance at a 3 : 1 (resistance : susceptibility) ratio indicating the respective presence of a single dominant resistance gene in ‘Ma851’ (Fraser-Smith et al., 2016). Three months after the initial inoculation, ‘p168’ showed minimal leaf symptoms, no stem splitting and a healthy rhizome (Figure 3a–c). In contrast, ‘p248’ showed necrotic lesions and the wilting of old leaves, a split stem and moderately discolored rhizome (Figure 3d–f). Confocal imagery of the inoculated resistant ‘p168’ line showed that the GFP was associated with the vascular bundles in the central cylinder toward the lower part of the rhizome (Figure 4A,B). GFP fluorescing hyphae was present in the cortex region near the xylem perforation plates (Figure 4B). Furthermore, patches of mycelial networks were detected at a low rate in the cortex region of ‘p168’ rhizome (Figure 4C). In contrast, mycelia associated with a strong GFP signal was detected in the mid and lower regions of the ‘p248’ rhizome where black discoloration was observed (Figure 4D,E).

The GFP signal of GFP-*Foc*-STR4 is strongly associated with the xylem perforation plates. Hyphal structures containing GFP were clearly observed in the cortex cells surrounding the vascular bundles (Figure 4D,E). The GFP-*Foc*-STR4 was strictly localized to the vascular bundles of the xylem and in the surrounding region (Figure 4F) suggesting that the fungus moves via the xylem. The cortex region of the uninoculated control showed little to no GFP fluorescence (Figure 4G). The xylem vessels of the uninoculated control were observed under the transmitted light setting (Figure 4H) and when stained with propidium iodide, they were associated with red-fluorescence at 555 nm (Figure 4I).

**Figure 5:**
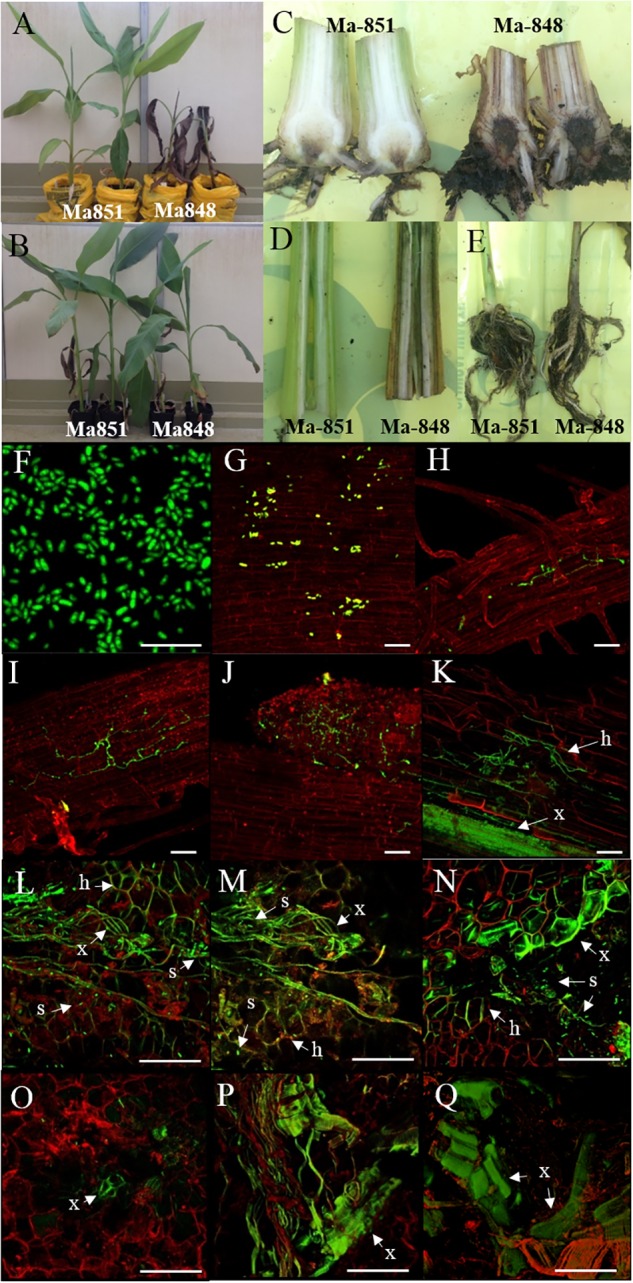
Characterization of *Foc*-race 4 resistance from *Musa acuminata* subsp *malaccensis* using a GFP tagged *Foc*-STR4 isolate (BRIP 23598, VCG 0120). **(A)** Plant phenotypes of the resistant ‘Ma851’ and susceptible ‘Ma848’ *M. a malaccensis* plants at 28 days post-inoculation (dpi). **(B)** Non-inoculated controls of ‘Ma851’ plants (left) and ‘Ma848’ plants (right). Longitudinal sections of **(C)** rhizomes, **(D)** pseudostem and **(E)** entire roots of ‘Ma851’ and ‘Ma848’ at 28 days dpi, respectively. **(F)** Spores collected from suspension culture of BRIP23598 after 5 days of growth in half strength PDB containing 50 mg per L of hygromycin B. **(G–K)** Visualization of the GFP protein on ‘Ma848’ under a confocal microscope. **(G)** Attachment of spores to the lateral root surface at 1 dpi. **(H)** Movement of hyphae in the epidermis layer of a lateral root at 2 dpi. **(I)** A mycelial network established on the epidermis at 4 dpi. **(J)** The apical meristem region of a root tip completely colonized by GFP-*Foc*-STR4 at 7 dpi. **(K)** Mycelial networks are established in the cortex and vascular bundles of the xylem at 14 dpi. The **(L)** upper, **(M)** mid and **(N)** lower sections of the rhizome from a ‘Ma848’ plant at 14 days dpi. The **(O)** upper, mid **(P)** and lower **(Q)** sections of the rhizome from a ‘Ma851’ plant at 14 days dpi. White bars indicate a 50 μm scale. GFP fluorescence was detected at 488 nm wavelength using a Zeiss 700 laser scanning microscope. The tissues were stained with propidium iodide to produce a red fluorescence which was detected at 555 nm wavelength. x = xylem vessels, s = individual or clumps of spores, h = hyphae.

Although no internal symptoms were detected in the resistant genotype ‘p168,’ the presence of GFP-*Foc*-STR4 in its rhizome suggests that resistance does not inhibit the pathogen from gaining entry into its roots. To further evaluate the resistance response, we used two of the *Musa acuminata* ssp. *malaccensis* parental lines, one that has been shown to be resistant, ‘Ma851,’ and one susceptible, ‘Ma848,’ against both *Foc*-STR4 and *Foc*-TR4 (Fraser-Smith et al., 2016). Progeny of ‘Ma851’ segregates for *Foc* resistance (Figure 3) whereas ‘Ma848’ showed an extremely susceptible phenotype indicative of the absence of any resistance genes (Figure 1, 2). In this assay, a *Foc* spore suspension and a root dipping inoculation method was used which has been reported previously to study the onset of the infection process (Li et al., 2011; Zhang et al., 2018). The inoculation rapidly induced wilting in the susceptible ‘Ma848’ lines with the first signs of wilting observed at 2–3 weeks post-inoculation (Supplementary Figure 3). ‘Ma848’ plants were observed to be dead at 4 weeks (Figure 5A). Inoculated ‘Ma851’ and un-inoculated controls of both lines did not show any external symptoms at 4 weeks (Figure 5A,B). When cut in halves, the rhizomes of ‘Ma848’ showed extensive discoloration as typically associated with necrotic lesions of vascular vessels (Figure 5C). The symptoms also include the discoloration of the pseudostem and a root structure of reduced size (Figure 5D,E). While ‘Ma851’ showed some noticeable discoloration in the lower part of the rhizome, discoloration in the pseudostem was not observed (Figure 5C,D).

To study the infection process, the presence of GFP-*Foc*-STR4 in ‘Ma848’ and ‘Ma851’ was visualized with confocal microscopy. The GFP tagged *Foc*-STR4 spores were confirmed to be constitutively fluorescing prior to the start of the experiment (Figure 5F). Adhesion of GFP-*Foc*-STR4 spores to the lateral roots of ‘Ma848’ was observed at 1 day post-inoculation (dpi, Figure 5G). The presence of hyphae on the epidermis of lateral roots of ‘Ma848’ was first observed at 2 dpi (Figure 5H). A longitudinal section of a lateral root shows the elongation of hyphae and the establishment of a mycelial network on the epidermis of lateral roots of ‘Ma848’ at 4 dpi (Figure 5I). Complete colonization of a lateral root tip by GFP-*Foc*-STR4 was observed in ‘Ma848’ at 7 dpi (Figure 5J). The presence of GFP-*Foc*-STR4 was not detected on the main roots of ‘Ma848’ during the first 7 dpi. A single hypha was observed attempting to penetrate the epidermis cell layer of the lateral roots in ‘Ma848’ at 7 dpi (Supplementary Figure 4A). Presence of hyphae was also detected on the epidermal cell layer of the fine roots in ‘Ma848’ (Supplementary Figure 4B). The presence of GFP-*Foc*-STR4 was not detected in the roots of the resistant line ‘Ma851’ (Supplementary Figures 4C–E). At 14 dpi, GFP tagged mycelium was observed in the root cortex region of ‘Ma848’ (Figure 5K). The xylem vessels also showed a strong GFP signal indicating the presence of the fungus within the xylem. The rhizome was sectioned into three parts, which include the upper region closer to the pseudostem, the middle region which is the central cylinder and the lower region that connects to the cortex and the roots. At 14 dpi, all three regions of the ‘Ma848’ rhizome showed the abundant presence of spores near the xylem perforation plates which were also infected with GFP-*Foc*-STR4 (Figure 5L–N). GFP-*Foc*-STR4 can be seen colonizing the cortex cells around the xylem vessels in ‘Ma848.’ In contrast, no spores were observed in the rhizome of the inoculated resistant ‘Ma851’ plants (Figure 5O–Q). Movement of hypha was also not observed. However, strong GFP fluorescence was observed in the xylem vessels, indicating the likely presence of GFP-*Foc*-STR4 in these vessels (Figure 5O–Q). These data suggest that the frequency of fungal penetration via the roots was greatly induced in ‘Ma851.’

### Isolation of GFP-*Foc*-STR4 Using Koch’s Postulates

Susceptible ‘p96’ and resistant ‘p3,’ ‘p18,’ ‘p104’ self-derived progeny of ‘Ma851’ were inoculated with GFP-*Foc*-STR4 and grown in a glasshouse for 3 months. At harvest, internal and external symptoms of *Fusarium* wilt were visible on the ‘p96’ plants (Supplementary Figures 5, 6). Extensive leaf wilting, corm rot and stem splitting were present in the susceptible ‘96’ plant but not in the resistant plants. Some slight discoloration of the corm was evident in the resistant plants (Supplementary Figure 5).

The presence of GFP-*Foc*-STR4 was detected in most of the regions isolated from the susceptible ‘p96’ plant, which include the throat and the mid-stem region of stem, and the mid-corm and cortex region of the corm (Supplementary Figures 7, 8 and Table 1). GFP-*Foc*-STR4 was not detected at all in two resistant ‘p3’ and ‘p18’ plants. However, it was isolated with the presence of the GFP confirmed in the regions of the throat in the third resistant ‘p104’ plant (Supplementary Figures 7, 8 and Table 1).

## Discussion

*Fusarium* wilt is one of the major threats confronting the banana industry today. An effective long-term solution to this problem would be to identify genetic resistance from the untapped gene pools in the wild relatives of the cultivated banana and introduce the identified sources of resistance loci back into commercially viable varieties. Resistance screens are traditionally performed in the field, which is labor-intensive owing to the long growth cycle and the large size of banana plants. In this study, a glasshouse screen method was adopted to assess resistance level in relatively young plants grown in pots. This type of assay can have a short turn-over time due to the up-scaling of the number of plants that can be tested at a given time under controlled conditions with minimized cross infection by other micro-organisms (Smith et al., 2008; Li et al., 2015; Zuo et al., 2018). In addition, high inoculum dosage can be applied to identify highly resistant germplasm.

Resistance against *F. oxysporum* f. sp. *cubense* was assessed in a collection of 34 genotypes of diploid and polyploid banana plants in the glasshouse (Table 1) and their resistance levels were ranked using the internal discoloration of the rhizome. A wide range of disease responses from completely resistant, to partially resistant and highly susceptible, was revealed in the plant rhizomes against *Foc* race 4 types (Figure 1, 2). Resistance levels of the genotypes tested were mostly consistent with previously published field data (Table 1). This response shows that resistance to *Foc* race 4 is mainly of a quantitative nature. Quantitative resistance has been detected in other pathosystems including *Pisum sativum* and *Medicago truncatula* against *Fusarium oxysporum* f. sp. *pisi* and *Fusarium oxysporum* f. sp. *medicaginis*, respectively (Bani et al., 2012; Rispail and Rubiales, 2014). In this study, wilt resistance can be detected in each of the diploid, triploid and tetraploid genome groups and in different *Musa accuminata* ssp. *malaccensis*, *Musa accuminata* ssp. *burmannica*, *Musa accuminata* ssp. *banksia*.

The *M. acuminata* ssp. *malaccenis* line ‘Ma851’ appears to carry strong wilting resistance to *Foc*. Wild *M. acuminata* diploids are highly diversified and have been heavily integrated in the breeding of edible banana cultivars (Perrier, 2009; Perrier et al., 2011; Li et al., 2013; Christelova et al., 2017). Geographically *M. acuminata* sub-species shows a wide-spread pattern of distribution in East Asia with each sub-species localized in distinct regions (Perrier et al., 2011). This suggests that some of these diploids may have evolved different mechanisms of resistance independently of one another. Similar findings were obtained in other studies, particularly in *Medicago truncatula* and *Pisum sativum*, which showed that quantitative resistance in their respective collections corresponds to accessions originating from multiple locations (Grünwald et al., 2003; Rispail and Rubiales, 2014). Future work will aim to identify QTLs controlling resistance in *M. a. malaccensis* lines.

The screen for *Foc* race 4 resistance revealed that the collection of *Musa* spp. contained sufficient genetic variations for the resistance responses to be detected. Cultivars shown to be highly susceptible to *Foc* race 4 in the field showed strong necrotic lesions in the rhizomes under our conditions (Table 1). In our study, the extent of the discoloration also depends on the inoculation technique used as it may influence the inoculum dosage and thereby the rate of the infection process. This observation is supported by studies that show a positive correlation between the amount of *F. oxysporum* detected within plant tissue and the resistance level in several species, including pea, tomato, watermelon, and chickpea (Gao et al., 1995; Zhou and Everts, 2004; Jimenez-Fernandez et al., 2011; Rispail and Rubiales, 2014).

In banana, contrasting resistance responses against *Foc* race 4 types have not been previously reported. In the present study, two cultivars, namely ‘FHIA-2’ and ‘CAM020’ showed excellent resistance to *Foc*-TR4 but were highly susceptible to *Foc*-STR4 (Figure 1, 2). This indicates that resistance to the two race 4 type VCGs might be differentially regulated. One possible explanation is that both cultivars lack a gene(s) or a component of the PAMPs (pathogen-associated molecular patterns) triggered immunity (PTI) or the effector-triggered immunity (ETI) that is specifically required for *Foc*-STR4 mediated resistance in these lines. Mechanisms of PTI and ETI are discussed in detail in recent reviews (Jie and Zhou, 2010; Petit-Houdenot and Fudal, 2017). Furthermore, both *Foc* race 4 type resistance can potentially be controlled by a single gene. For example, it has been shown in tomato that the immune receptor Ve1 mediates resistance against multiple pathogens by recognizing not only the *Verticillium* effector *Ave1*, but also related homologs of *Ave1*, from *Fusarium oxysporum* f. sp. *lycopersici* and *Cercospora beticola* (de Jonge et al., 2012).

In soil borne plant diseases, such as those caused by *Foc*, where the pathogen gains entry via plant roots, the state of the rhizome is a good indicator of the severity of infection. Necrotic lesions in the rhizome are the consequences of *Foc* colonizing the vascular tissues to cause senescence in a localized manner. This was evident in the rhizomes of the susceptible banana plants used in this study (Figure 3, 4). As shown using GFP tagged *Foc*-STR4, mycelial networks typically migrated along the xylem vessels and expanded outwards from the pits in susceptible plants. Our results are consistent with the behavior and the strong virulence of *Foc* race 4 observed in susceptible banana cultivars (Li et al., 2011, 2017; Zhang et al., 2018). A high level of sporulation further supports this observation in the present study (Figure 4).

In our study, the resistant plants typically showed no discoloration when millet was used as the inoculum (Figure 1–3). Furthermore, fungal isolation performed on rhizome and stems tissues of the resistant plants 3 months post-millet inoculation also failed to recover the GFP-STR4 strain (Supplementary Table 1). This suggests that the frequency of fungal colonization was too low to be detected in these lines at the respective early and late stages. This finding is consistent with a previous study which showed that *Foc* was not detectable at an early stage of infection in resistant banana plants (Li et al., 2011). A possibility could be that the growth of *Foc* was inhibited. Root exudates can potentially inhibit spore germination and fungal growth. In pea, phytoalexin pisatin, one of the metabolites detected in the root exudate extracts of pea, negatively correlated with the extent of *Fusarium oxysporum* f. sp. *pisi* germination (Bani et al., 2018a). It is of note that the millet inoculation technique minimized root wounding, whereas artificially induced wounding can enhance the infection rate in the roots and cause GFP tagged *Foc* to be detected in the rhizome of resistant banana cultivar Pahang (Zhang et al., 2018). However, in our study, *Foc* was never-the-less inhibited from traveling further up the pseudo-stem of the plant.

When a relatively more invasive method, root dipping, and concentrated micro-conidia were applied, GFP signals were not detected in the roots of resistant ‘Ma851’ plants at 7 dpi (Supplementary Figure 4). However, necrotic lesions were observed in the rhizome of the resistant plants at 18 dpi (Supplementary Figure 3). At this time, GFP fluorescence was associated with the xylem perforation plates. However, no sporulation or mycelial networks were observed (Figure 4). This suggests that the colonization by the fungus was mainly contained in the rhizome of ‘Ma851.’ Furthermore, no GFP-*Foc*-STR4 was detected in the stems of ‘Ma851’ suggesting that the xylem was likely uninfected (Supplementary Table 1). A similar pattern of restricted colonization in *Dianthus caryophyllus* by *Fusarium oxysporum* f. sp. *dianthi* has been reported and further characterization revealed that the infected regions of the xylem became compartmentalized by cell wall thickening and hyperplasia of parenchyma cells (Ouellette et al., 1999).

This type of resistance mechanisms against vascular pathogens have been characterized in several plant species (Beckman et al., 1982; Bishop and Cooper, 1983a,b; Baayen et al., 1989; Tessier et al., 1990; Pereira et al., 2013; Bani et al., 2018b). Plant hosts develop physical and chemical barriers to block pathogen progressions at different stages during the infection process. These include cell wall strengthening by lignification and suberization, formation of papillae at penetration sites, the accumulation of tyloses inside cells and production of antifungal compounds (Beckman et al., 1982; Bishop and Cooper, 1983a; Baayen and Elgersma, 1985; Charchar and Kraft, 1989; Ouellette et al., 1999; Grayer and Kokubun, 2001; Yadeta and Thomma, 2013; Pouralibaba et al., 2017; Bani et al., 2018b). Beckman et al. (1982) showed that *F. oxysporum* triggered callose deposition in the parenchyma cells of tomato plants and that the rate of deposition was faster in the resistant than the susceptible plants. In a separate experiment, Robb et al. (1991) showed that *Verticillium* infected tomato petioles induced suberization in the membranes of the pits and the intercellular spaces around the vascular vessels. Furthermore, the level of vascular coating positively correlated with resistance and negatively correlated with the frequencies of pathogen penetration of pit membranes in alfalfa (Newcombe and Robb, 1988). One or any of these mechanisms could potentially explain the resistance mechanism observed in this study.

In the present study, we have assessed a collection of 34 banana cultivars for resistance against *F. oxysporum* f. sp. *cubense* race 4 types and identified a range of resistance responses in the rhizomes. The rhizome appears to be a key factor in preventing the fungus from further spreading to other parts of the plant. We characterized diploid wild relative *M. a. malaccensis* lines that exhibit strong wilt resistance to *Foc* by inhibiting fungal growth in its rhizome. They are potential sources of ‘complete’ resistance to *Foc* race 4. Furthermore, contrasting resistance responses to different *Foc* race 4 types were observed. Phenotypic methods used in this study can help accelerate the efforts in breeding programs. Overall, this study paves the way for further characterizations in the defense mechanisms of *Foc* resistance at the cellular and molecular level in this important plant species.

## Author’s Note

Plants used in this study were generated from the Australian in vitro banana cultivar collection that is maintained in the Quality Banana Approved Nursery (QBAN) scheme accredited Plant Biotechnology Laboratory located at the Maroochy Research Facility, Department of Agriculture and Fisheries, Nambour, Queensland, Australia. The cultivars were directly sourced from the owners or institutions under verbal or written agreement and where plants were directly collected, such as *Musa acuminata* ssp. *malaccensis* lines, they were sourced prior to the development of International treaties for germplasm. All cultivars were sourced under agreements allowing that they are able to be used for research purposes. No ownership is claimed for those cultivars sourced from owners or other institutions. The plants were destructively sampled for the purposes of this experiment and will not be further propagated.

## Author Contributions

AC, SH, JB, and EA proposed, organized, and planned the experiments. AC, JS, AM, LA-E, NC, SM, and LT-N carried out and performed the experiments. AC wrote the manuscript draft. All authors commented and contributed to the preparation of the final manuscript.

## Conflict of Interest Statement

The authors declare that the research was conducted in the absence of any commercial or financial relationships that could be construed as a potential conflict of interest.

## Abbreviations

dpi: days post-inoculation
*Foc*: *Fusarium oxysporum* f sp. *cubense*
FHIA: Fundacion Hondurena de Investigación Agrícola
GFP: green fluorescent protein
Ma: *Musa accuminata* ssp. *malaccensis*
SH: synthetic hybrid
STR4: subtropical race 4
TR4: tropical race 4
VCG: vegetative compatibility group.
