# Supplementary material for: Assessing Variations in Host Resistance to Fusarium oxysporum f sp. cubense Race 4 in Musa Species, With a Focus on the Subtropical Race 4
Source: Front Microbiol. 2019 May 15;10:1062. doi: 10.3389/fmicb.2019.01062 (PMC6529558; doi:10.3389/fmicb.2019.01062)
Supplement: Supplementary file 1 [file Data_Sheet_1.docx]

**Supplementary Materials**

**Assessing variations in host resistance to *Fusarium oxysporum* f sp. *cubense* race 4 in *Musa* species.**

**Andrew Chen^1^*, Jiaman Sun^2^, Andrea Matthews^1^, Liz Armas-Egas^1^, Ning Chen^1^, Sharon Hamill^3^, Sharl Mintoff^4^, Lucy T. T. Tran-Nguyen^4^, Jaqueline Batley^1,5^, Elizabeth A. B. Aitken^1^.**

**^1^ School of Agriculture and Food Science, The University of Queensland, St Lucia, Brisbane, QLD, Australia.**

**^2^ Guangxi Crop Genetic Improvement and Biotechnology Key Lab, Guangxi Academy of Agricultural Sciences, Nanning, China**

**^3^ Department of Agriculture and Fisheries, Maroochy Research Facility, Nambour, QLD, Australia.**

**^4^ Department of Primary Industry and Resources, Northern Territory Government, Darwin, NT, Australia**

**^5^ School of Biological Sciences, The University of Western Australia, Perth, WA, Australia.**

***Correspondence:** Dr Andrew Chen: [a.chen2@uq.edu.au](mailto:a.chen2@uq.edu.au)

**Supplementary Data**

**Supplementary Table 1.** **Assessment of fungal colonies grown from primary isolations of *M. a. malaccensis* plant tissues infested with GFP-*Foc*-STR4.**

**Supplementary Figure 1.** **Assessment of internal symptoms in the rhizomes of genotypes challenged with *Foc*-SR4 (VCG 0120, isolates BRIP63488, BRIP43781, BRIP42331).**

**Supplementary Figure 2. Assessment of internal symptoms in the rhizomes of genotypes challenged with a *Foc*-TR4 VCG 01213/16 isolate.**

**Supplementary Figure 3.** **GFP tagged *Foc*-SR4 infested *M. a. malaccensis* plants ‘Ma851’ and ‘Ma848’ at 18 days post inoculation.**

**Supplementary Figure 4.** **Confocal imagery of GFP tagged *Foc*-SR4 observed on the roots of *M. a. malaccensis* plants ‘Ma848’ and ‘Ma851’ at 7 days post inoculation.**

**Supplementary Figure 5. *Fusarium* wilting symptoms of the three resistant (p3, p18 and p104) and one susceptible (p96) plants, inoculated using the millet method, at three months post inoculation.**

**Supplementary Figure 6.** **The susceptible ‘Ma851’ progeny plant p96 displayed discolouration in a longitudinal section of the rhizome, specifically in the regions where the root nodes join the rhizome.**

**Supplementary Figure 7.** **Primary isolates (Koch’s postulates) obtained from tissues of the ‘Ma851’ progeny plants p3, p18, p104 and p96.**

**Supplementary Figure 8.** **Images of GFP fluorescence detected in the liquid cultures of the fungal isolates shown in Supplementary Figure 7.**

| Plant | Isolation | Primary Growth | *Foc*-like spores | Growth in PDB + H | GFP Fluorescence |
| --- | --- | --- | --- | --- | --- |
| Plant 104 (resistant) | A1 | - |  |  |  |
|  | **A2** | **+** | **+** | **+** | **+** |
|  | **A3** | **+** | **+** | **+** | **+** |
|  | A4 | - |  |  |  |
|  | B1 | - |  |  |  |
|  | B2 | - |  |  |  |
|  | B3 | - |  |  |  |
|  | B4 | - |  |  |  |
|  | C1 | - |  |  |  |
|  | C2 | - |  |  |  |
|  | C3 | - |  |  |  |
|  | C4 | - |  |  |  |
|  | D1 | - |  |  |  |
|  | D2 | - |  |  |  |
|  | D3 | - |  |  |  |
|  | D4 | - |  |  |  |
|  | E1 | - |  |  |  |
|  | E2 | - |  |  |  |
|  | E3 | - |  |  |  |
|  | E4 | - |  |  |  |
| Plant 3 | A1 | - |  |  |  |
| (resistant) | A2 | - |  |  |  |
|  | **A3** | **+** | **-** | **-** |  |
|  | A4 | - |  |  |  |
|  | B1 | - |  |  |  |
|  | B2 | - |  |  |  |
|  | B3 | - |  |  |  |
|  | B4 | - |  |  |  |
|  | C1 | - |  |  |  |
|  | C2 | - |  |  |  |
|  | C3 | - |  |  |  |
|  | C4 | - |  |  |  |
|  | D1 | - |  |  |  |
|  | D2 | - |  |  |  |
|  | D3 | - |  |  |  |
|  | D4 | - |  |  |  |
|  | E1 | - |  |  |  |
|  | E2 | - |  |  |  |
|  | E3 | - |  |  |  |
|  | E4 | - |  |  |  |
| Plant 18 | **A1** | **+** | **-** | **-** |  |
| (resistant) | A2 | - |  |  |  |
|  | A3 | - |  |  |  |
|  | **A4** | **+** | **-** | **-** |  |
|  | **B1** | **-** |  |  |  |
|  | **B2** | **+** | **-** | **-** |  |
|  | **B3** | **+** | **-** | **-** |  |
|  | B4 | - |  |  |  |
|  | C1 | - |  |  |  |
|  | C2 | - |  |  |  |
|  | C3 | - |  |  |  |
|  | C4 | - |  |  |  |
|  | D1 | - |  |  |  |
|  | D2 | - |  |  |  |
|  | D3 | - |  |  |  |
|  | D4 | - |  |  |  |
|  | E1 | - |  |  |  |
|  | E2 | - |  |  |  |
|  | E3 | - |  |  |  |
|  | E4 | - |  |  |  |
| Plant 96 | **A1** | **+** | **+** | **+** | **+** |
| (susceptible) | **A2** | **+** | **+** | **-** |  |
|  | A3 | - |  |  |  |
|  | A4 | - |  |  |  |
|  | **B1** | **+** | **+** | **-** |  |
|  | B2 | - |  |  |  |
|  | **B3** | **+** | **-** | **-** |  |
|  | **B4** | **+** | **-** | **+** | **+** |
|  | C1 | - |  |  |  |
|  | C2 | - |  |  |  |
|  | **C3** | **+** | **-** | **+** | **-** |
|  | **C4** | **+** | **-** | **+** | **-** |
|  | **D1** | **+** | **+** | **-** |  |
|  | **D2** | **+** | **-** | **+** | **+** |
|  | **D3** | **+** | **-** | **+** | **+** |
|  | **D4** | **+** | **+** | **+** | **+** |
|  | **E1** | **+** | **+** | **-** |  |
|  | **E2** | **+** | **+** | **-** |  |
|  | **E3** | **+** | **+** | **+** | **+** |
|  | **E4** | **+** | **-** | **+** | **+** |

**Supplementary Table 1.** **Assessment of fungal colonies grown from primary isolations of *M. a. malaccensis* plant tissues infested with GFP-*Foc*-STR4.**

Colonies were subcultured to potato dextrose broth containing 50 mg per L hygromycin B. In the isolation column, the alphabet A to E indicates the five regions from which isolation was performed. These include A, the upper stem just below the first leaf petiole (throat), B, mid-point of the stem, C, stem just above the rhizome, D, the central cylinder of the rhizome, and E, the outer layer of the rhizome connecting to the cortex. The number 1 to 4 immediately following the letters indicate the four pieces of tissues isolated in each region. Fungal morphology and the presence of *Foc*-like spores were checked under a dissecting microscope. Presence of GFP-*Foc*-STR4 mycelium was visualised using the confocal microscope.


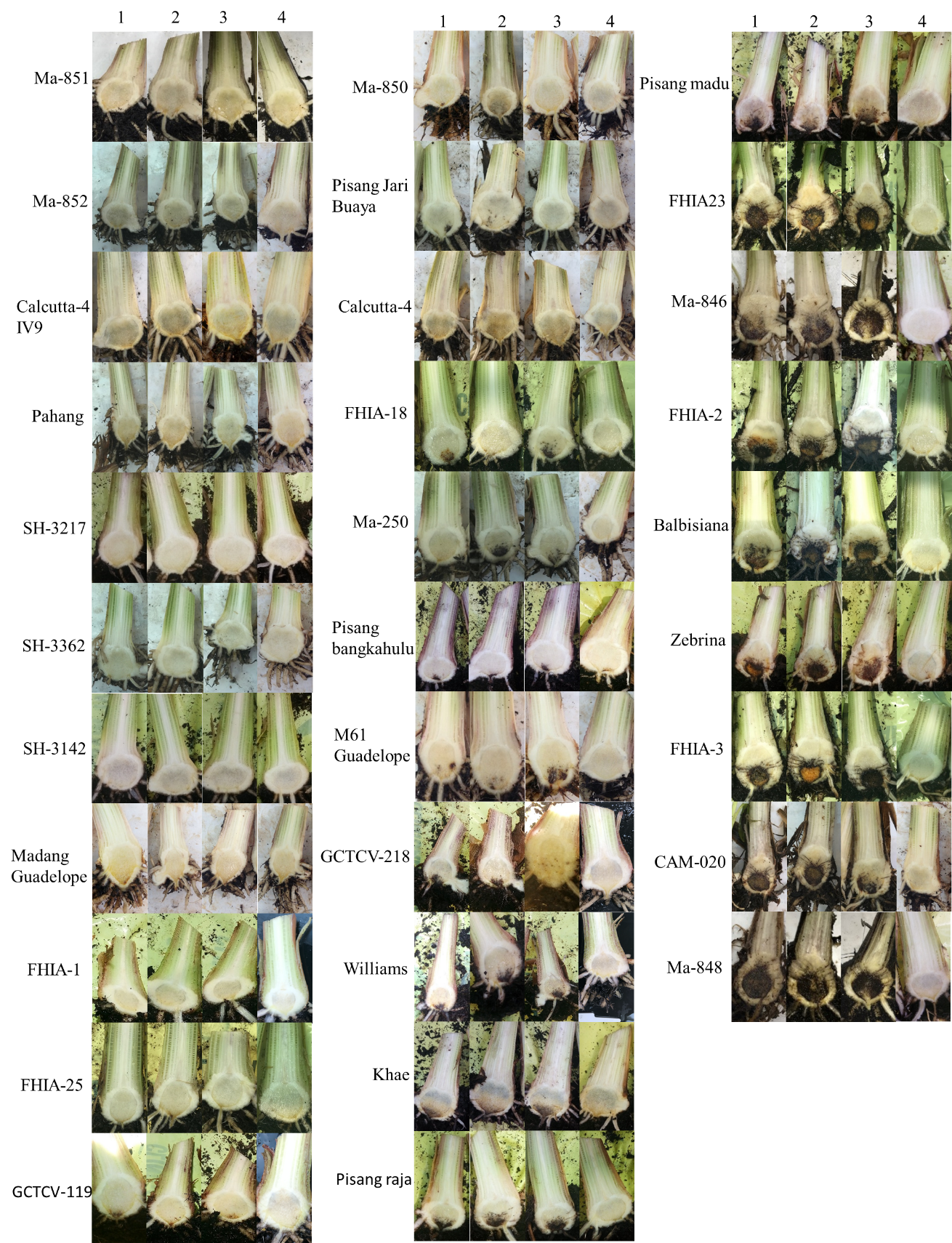


**Supplementary Figure 1.** **Assessment of internal symptoms in the rhizomes of genotypes challenged with *Foc*-SR4 (VCG 0120, isolates BRIP63488, BRIP43781, BRIP42331).**

Each isolate was prepared on half strength PDA plates and 3 to 4 agar plugs from fully grown plates were used to inoculate millet. Millet colonised with each of the three isolates was mixed in equal amounts and 40 grams of this mixture inoculum was applied to the soil in 200 mm diameter pots. Symptoms were assessed 12 weeks post inoculation. Rhizomes were cross-sectioned and discoloration in the stelar regions was assessed and then scored using a 1 to 8 scale previously published (Mak et al., 2004).


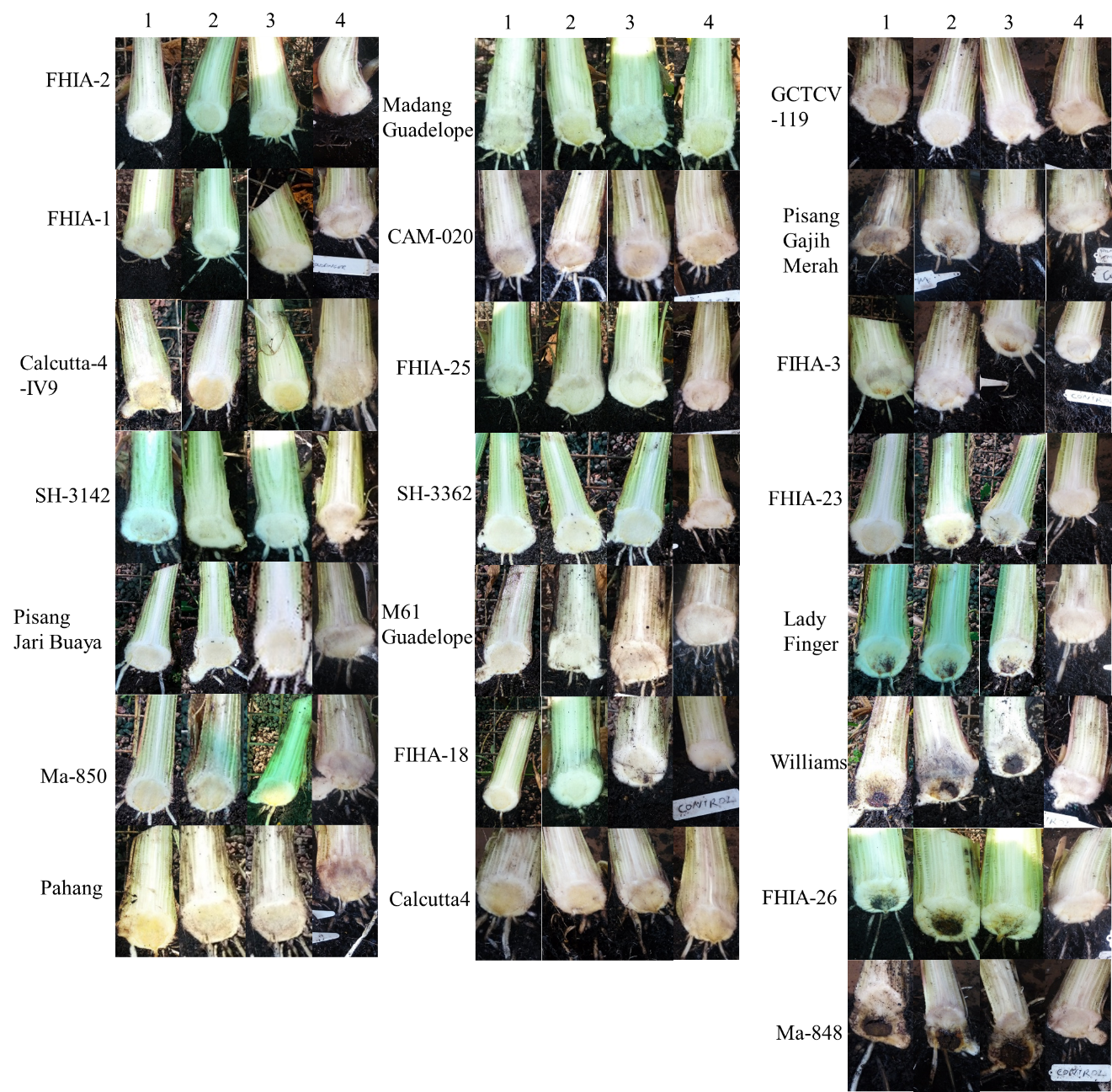


**Supplementary Figure 2. Assessment of internal symptoms in the rhizomes of genotypes challenged with a *Foc*-TR4 VCG 01213/16 isolate.**

This isolate was prepared on half strength PDA plates and 3 to 4 agar plugs from fully grown plates were used to inoculate millet. Millet colonised with each of the 4 isolates was mixed in equal amounts and 30 to 50 grams of this mixture inoculum was applied to the soil in 200 mm diameter pots. Symptoms were assessed 12 weeks post inoculation. Rhizomes were cross-sectioned and discoloration in the stellar regions was assessed and then scored using a 1 to 8 scale previously published (Mak et al., 2004).


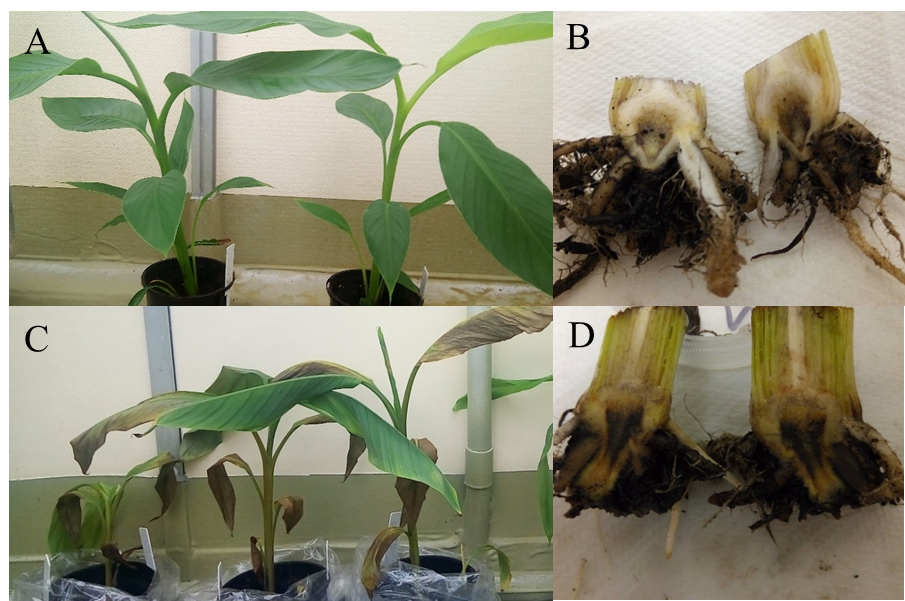


**Supplementary Figure 3.** **GFP tagged *Foc*-SR4 infested *M. a. malaccensis* plants ‘Ma851’ and ‘Ma848’ at 18 days post inoculation.**

Individuals of **(A)** ‘Ma851’ and **(B)** ‘Ma848’ in pots with the ‘Ma848’ plant showing a severe wilting phenotype. **(C)** Longitudinal sections of the rhizome of the ‘Ma851’ plant. (**D)** Longitudinal sections of the rhizome of the ‘848’ plant.


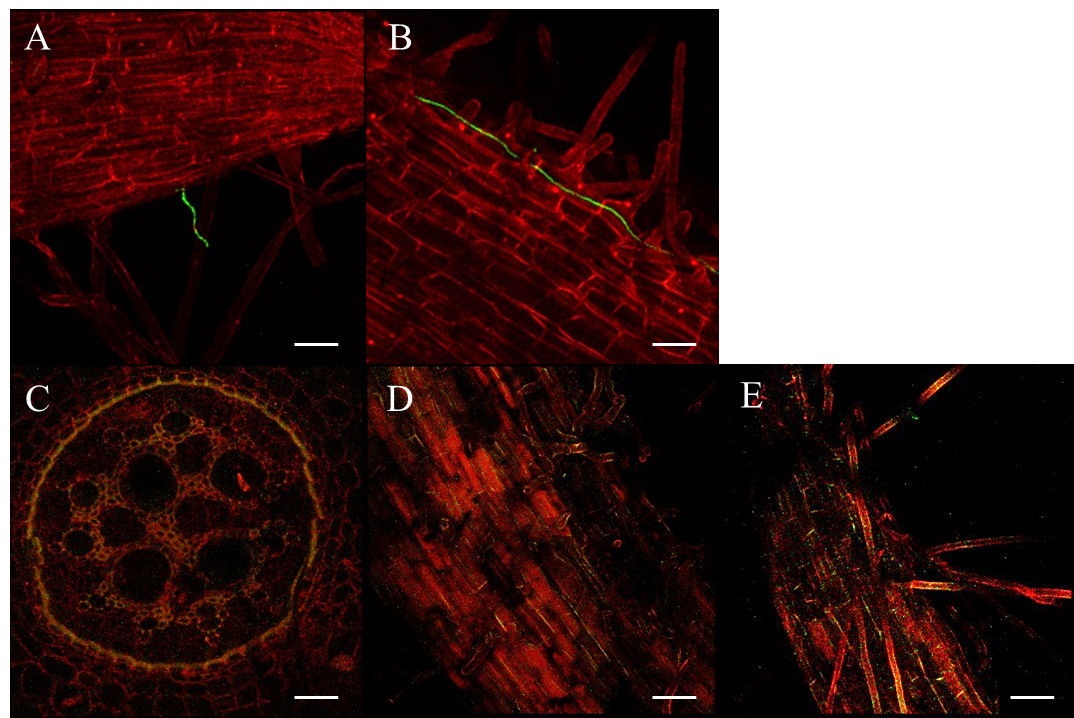


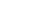

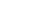


**Supplementary Figure 4.** **Confocal imagery of GFP tagged *Foc*-SR4 observed on the roots of *M. a. malaccensis* ‘Ma848’ and ‘Ma851’ plants 7 days post inoculation.**

**(A)** Attachment of mycelium on the fine root hairs and its attempt to penetrate the surface of a lateral root of ‘Ma848’. **(B)** Movement of the mycelium on the epidermal layer of a lateral root of ‘Ma848’. **(C)** A cross section of ‘Ma851’ lateral root showing the absence of GFP in its vascular vessels and the surrounding cortex area. **(D)** Epidermis of a lateral root of ‘Ma851’. **(E)** Root hairs of lateral root of ‘Ma851’. Root tissues are stained with 10ug per mL Propidium Iodide before visualisation under the confocal microscope. Scale bars = 50µm.


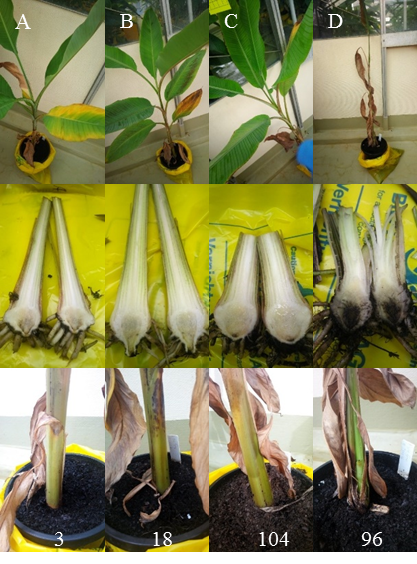


**Supplementary Figure 5. *Fusarium* wilting symptoms of the three resistant (p3, p18 and p104) and one susceptible (p96) plants, inoculated using the millet method, at three months post inoculation.**

The individuals were selected from an F_2_ population, derived from a self-cross of the *M. a. malaccensis* plant 851. Images were taken just before the preparation of samples for Koch’s postulates. **(A, B, C, D)** indicates the respective whole plant, rhizome and stem of each individual at the time of harvest.


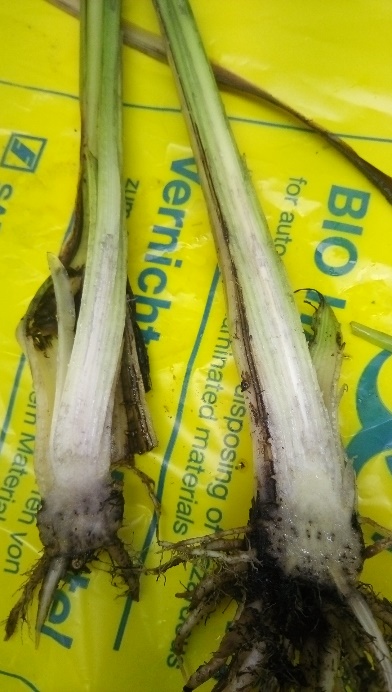


**Supplementary Figure 6.** **The susceptible ‘Ma851’ progeny plant p96 displayed discolouration in a longitudinal section of the rhizome, specifically in the regions where the root nodes join the rhizome.**

The p96 progeny plant was inoculated with GFP-*Foc*-STR4 infested millet. The image was taken at 3 months post inoculation.


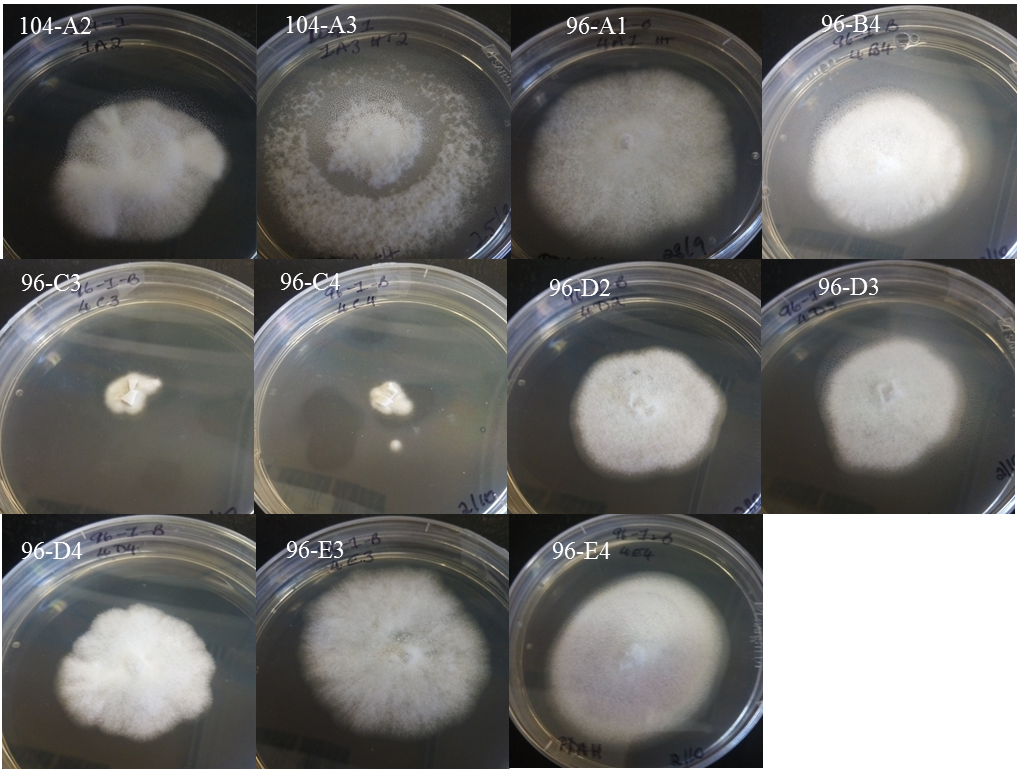


**Supplementary Figure 7.** **Primary isolates (Koch’s postulates) obtained from tissues of the ‘Ma851’ progeny plants p3, p18, p104 and p96.**

The isolates were subcultured on PDA containing 50 mg per L hygromycin B to verify the presence of hygromycin B-resistance containing plasmid in GFP tagged *Foc*-STR4. Fungal growth confirmed the presence of the resistance gene. The letters indicate the different regions of the plant from which the tissues were sampled for Koch’s postulates. A: the throat of the stem joining the leaves, B: vertically, the mid-point of the stem, C: the lower part of the stem joining the rhizome, D: middle region (central cylinder) of the rhizome, E: the outer region of the rhizome where cortex layer is located. The numbers 1-4 immediately following the letters indicate the individual replicates sampled for that particular region.


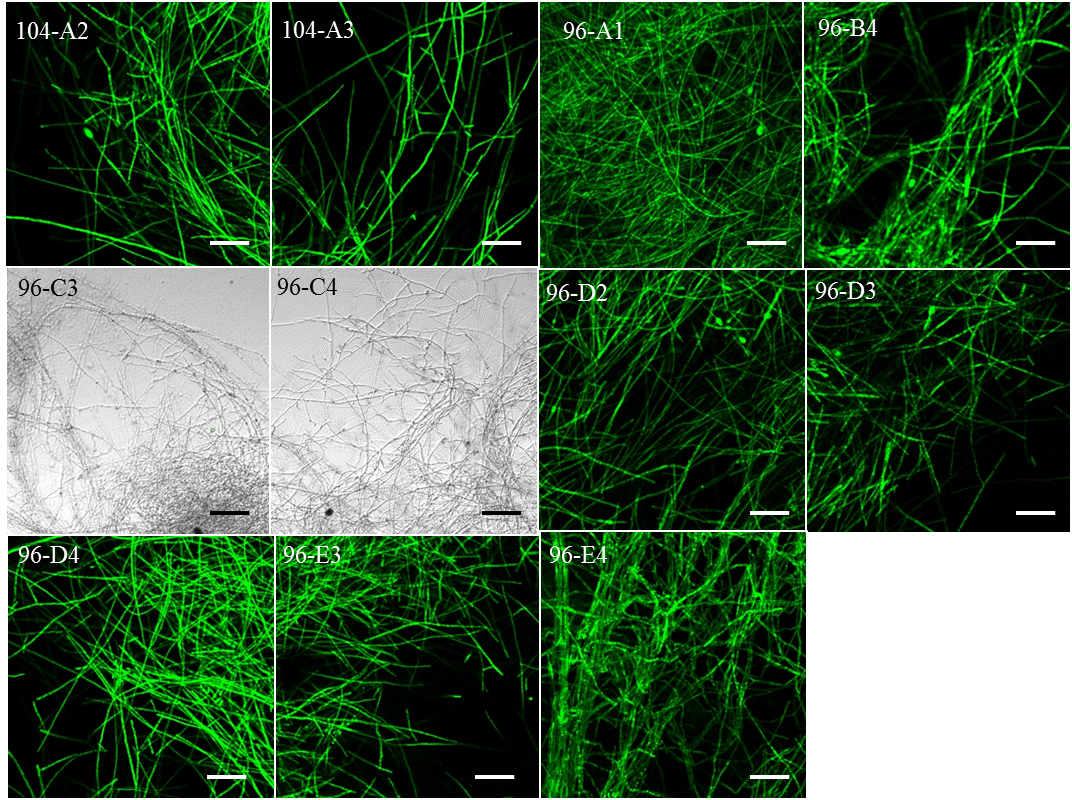


**Supplementary Figure 8.** **Images of GFP fluorescence detected in the liquid cultures of the fungal isolates shown in Supplementary Figure 7.**

The isolates were incubated in PDB amended with 50 mg per L hygromycin B. Visualisation was performed using the confocal microscope. Only light images were provided for cultures 96-C3 and 96-C4 as they grew poorly on PDA plates and did not produce fluorescence in PDB. Scale bars = 50µm. The letters indicate the different regions of the plant from which the tissues were sampled for Koch’s postulates. A: the throat of the stem joining the leaves, B: vertically, the mid-point of the stem, C: the lower part of the stem joining the rhizome, D: middle region (central cylinder) of the rhizome, E: the outer region of the rhizome where cortex layer is located. The numbers 1-4 immediately following the letters indicate the individual replicates sampled for that particular region.

1. Mak C, Mohamed AA, Liew KW, Ho YW. Early screening technique for Fusarium wilt resistance in banana micropropagated plants. In: Swennen R, Jain MS, editors. Banana Improvement: Cellular, Molecular Biology, and Induced Mutations. Enfield, New Hampshire, USA: Science Publishers, Inc, 2004. p. 219-27.
